# Conditions required to ensure successful detection and management of mild cognitive impairment in primary care: A Delphi consultation study in China

**DOI:** 10.3389/fpubh.2022.943964

**Published:** 2022-09-23

**Authors:** Yuan Lu, Chaojie Liu, Dehua Yu, Yvonne Wells

**Affiliations:** ^1^Department of General Practice, Yangpu Hospital, Tongji University School of Medicine, Shanghai, China; ^2^School of Psychology and Public Health, La Trobe University, Melbourne, VIC, Australia; ^3^Academic Department of General Practice, Tongji University School of Medicine, Shanghai, China; ^4^Shanghai General Practice and Community Health Development Research Center, Shanghai, China; ^5^Lincoln Center for Research on Aging, La Trobe University, Melbourne, VIC, Australia

**Keywords:** mild cognitive impairment, chronic care model, Delphi, primary care, China

## Abstract

**Objective:**

Detection and management of mild cognitive impairment (MCI) in primary care has been recognized internationally as one of the strategies that can be employed to delay the development of dementia. However, little is known about what role primary care should play. This study aimed to develop a checklist of conditions necessary for successfully detecting and managing mild cognitive impairment in primary care in China.

**Methods:**

This study employed the Delphi method to establish expert consensus on the conditions required for successfully detecting and managing MCI in primary care in China. Twenty-four experts who specialized in general practice, public health, neuropsychology, or community health service management rated the importance of pre-defined conditions (44 items measuring providers' preparedness, patient engagement, and system support in line with the Chronic Care Model). The degree of consensus among the experts was measured using four indicators: median ≥ 4, mean ≥3.5, Co-efficient of Variance < 0.25, and retention in the checklist required ≥ 80% agreement with a rating of important or essential. The checklist and descriptions of the conditions were revised according to the experts' feedback and then sent out for repeated consultations along with a summary of the results of the previous round of consultations. Consensus was achieved after the second round of consultations, which was completed by 22 of the experts.

**Results:**

The experts endorsed a checklist of 47 conditions required for successful detection and management of MCI in primary care in China. These conditions were categorized into four domains: prepared general practitioners (17 items), engaged patients (15 items), organizational efforts (11 items), and environmental support (4 items).

**Conclusions:**

Successful detection and management of MCI in primary care in China requires a dedicated and competent workforce of general practitioners, as well as the engagement of patients and family caregivers. Adequate support from healthcare organizations, health system arrangements, and the broader society is needed to enable effective interactions between general practitioners and patients and efficient delivery of the services required to detect and manage MCI.

## Objective

Mild cognitive impairment (MCI) is an intermediate phase between normal aging and dementia (1). Recognition that MCI may represent a transition state between normal cognitive decline due to aging and the development of dementia provides an opportunity for interventions to delay the onset of, or progression to, dementia (2). Nearly one-third of dementia cases could be delayed or prevented if early interventions were effectively adopted (3). The cost of implementing prevention strategies can be justified if they reduce future healthcare system costs. A study commissioned by the Alzheimer's Association in the US found early diagnosis could lead to better management of MCI and dementia and a potential cost savings of approximately $7 trillion, assuming that 88% of individuals who will develop Alzheimer's disease would be diagnosed in the MCI phase (4).

It has been recognized internationally that primary care has the potential to play an important role in detecting and managing MCI (5). In primary care-dominated health systems, general practitioners (GPs) are usually expected to manage chronic diseases due to their long-term relationship with patients. Control of most potentially modifiable risk factors for MCI is within the reach of GP services (3). Because of the convenience and continuity of their services, GPs are particularly well positioned to provide non-pharmacological interventions (6). They have the opportunity to observe any cognitive declines in their patients and can help patients to appreciate the value of preventive care. However, many cases of MCI are missed or neglected in primary care settings. For example, the Study on Aging, Cognition and Dementia in Primary Care Patients (AgeCoDe) in Germany shows that only around one-in-ten cases of MCI are recognized by GPs (7).

The underlying reasons for the under-recognition of MCI in primary care are likely to be multifaceted. The Chronic Care Model (CCM), one of the most widely adopted models guiding community management of chronic diseases, identifies eight elements of good practice (8): mobilizing community resources, enabling patient self-management, facilitating informal care support, promoting high-quality health system, improving health care delivery system, implementing decision support to meet the needs of health care providers, enhancing health care professional case management support, using patient/population data effectively. Empirical evidence shows that GPs often report insufficient time, limited knowledge and skills, and a lack of confidence as major barriers to detecting and managing MCI (9), while perceived stigma, low health literacy, and prioritization of other medical comorbidities often deter patients from seeking care for MCI (10).

In recent health reforms, the Chinese government attempted to revitalize its primary care system to confront the challenges of rapid population aging and the increasing burden of chronic illness. By 2021, 35,365 Community Health Centers (CHCs) had been established, with each covering a population of about 10,000 to 100,000 (11). A nationwide training program has been running since 2013 to supply skilled GPs to CHCs (12). The price of community health services has been set low to attract patients to choose CHCs as their first contact point of care (13), despite the absence of an official referral requirement (14). In addition, population-based per capita funding is available to support CHCs in delivering essential public health services, including the management of chronic diseases. Unfortunately, China does not yet have a national action plan on dementia (15), and the CHC funding for essential public health services does not cover dementia, nor MCI (16). Nevertheless, some local governments have determined to start building “friendly communities” for older people with cognitive impairment as part of their aged care strategy (17). Shanghai launched the first program of its kind in 2018, with the aim to coordinate community resources in supporting older adults with cognitive impairment through risk assessment, non-pharmacological interventions, family support, public education, information sharing, and coordination of social and medical services. CHCs were encouraged to provide MCI screening services. GPs were offered training relating to community detection and management of MCI, although a certificate awarded by such training is not mandatory for GPs to be involved in MCI screening and intervention.

Little is known about the conditions needed to enable successful detection and management of MCI in primary settings in China. Possible concerns include a workforce shortage of GPs in CHCs (18), and relatively low consumer trust in CHCs in comparison with hospitals (19). The current study addressed this gap in the literature by seeking expert consensus on the conditions required for successful detection and management of MCI in CHCs in China.

## Methods

### Study design

The Delphi method was adopted. The study design followed the “Guidance on Conducting and Reporting Delphi Studies (CREDES)” (20). Expert consensus was achieved through repeated consultations. Each expert was invited to rate the importance of a series of pre-defined conditions independently (21). They were also encouraged to suggest any changes, such as modification, deletion, or addition in open text boxes. The aggregated anonymous rating results were then fed back to the participants in subsequent consultations. This approach allowed participants to express their opinions free from peer pressure. The consultations were repeated until a consensus was reached among the participants.

The Delphi method is effective in establishing expert consensus when the published literature is not able to provide a robust conclusion. While expert consensus is deemed as a weak type of evidence in evidence-based medicine, its validity is supported by “wisdom of crowds:” research shows that groups can make good judgements under certain conditions (22). A recent systematic review suggests that a sample size in the low to medium double-digit range is adequate for a Delphi study (23). However, Delphi studies can now be run online with a very large panel size, as shown by Japan's NISTEP Delphi survey (24). Delphi method has been used in developing measures to assess the quality of primary care services in China (25). In the current study, the Delphi method was adopted because there is a paucity of literature documenting facilitators and barriers in community detection and management of MCI.

The study protocol was approved by the Human Ethics Committee of La Trobe University (HEC20125) and the Medical Ethics Committee of Yangpu Hospital, Shanghai, China (LL-2019-SCI-004).

### Delphi process

The Delphi consultations involved three stages: expert panel formation, development of consultation rating scales, and data collection and analysis (20).

#### Panel formation

Delphi panel members are intentionally selected to be diverse and “information rich” (26). Accordingly, in our study, participants were required to have a minimum of 10 years' work experience in any of the following areas: general practice, public health, neuropsychology, or community health service management. Eligible participants were identified through community health associations or were authors of peer-reviewed publications regarding community detection and management of MCI in China. An email invitation along with the informed consent letter and consultation rating scales were sent to the identified experts to solicit their interest in participation in this study. Return of the consultation rating scales was deemed as having provided informed consent.

In total, 24 experts participated in the study, covering all of the above-mentioned areas of experience.

#### Development of consultation rating scales

A focus group study involving GPs, people with MCI, their family caregivers, and CHC managers was used to gather stakeholders' perceptions on the detection and management of MCI to develop the consultation rating scales. Three themes were extracted from the focus group study: hesitant patients, unprepared providers, and misaligned environments. These themes were mapped to the following theoretical frameworks and translated into scale items under a three-level structure.

The overarching (first level) structure of the items was aligned with the CCM framework, which was developed in the 1990s (27) and has been widely applied to guide good practice in managing chronic diseases (28). It emphasizes the importance of prepared practice teams and well-informed and engaged patients, as well as a system platform that enables effective interactions between the two groups in managing chronic conditions. The themes generated from the focus group study covered all three above-mentioned domains.

The health belief model (HBM) guided the determination of the sub-domains (second level) in relation to “well-informed and engaged patients.” The HBM posits that patient behaviors are shaped by six elements: perceived susceptibility to getting a disease; perceived seriousness of the illness; potential benefits and barriers of a particular health action; confidence in one's ability to take action; and strategies to activate “readiness” (29). In the current study, factors associated with patient engagement were categorized into five sub-domains (30): patient-related (e.g., patient awareness and perceptions), illness-related (e.g., nature and trajectory of the illness condition), healthcare professional-related (e.g., the doctor-patient relationship and communication between doctors and patients), health care setting-related (e.g., infrastructure support), and task-related (e.g., interventional measures).

Four sub-domains (second level) in relation to “prepared practice teams” were identified corresponding to the Capability-Opportunity-Motivation-Behavior (COM-B) framework (31). The COM-B model posits that capability and opportunity are necessary conditions for volitional behavior to occur, while sufficient motivation energizes and directs the behavior. Capability was defined as an individual's physical and psychological ability to engage in the activity concerned, which includes factors such as knowledge, professional skills, and practice confidence. Opportunity was defined as the “factors that lie outside the individual that make the behavior possible or prompt it,” which can be physical (e.g., availability of time, funding, staff, tools) or social (e.g., social influence and social support) (31). Motivation was defined as an intrinsic process (such as a sense of purpose and autonomy) that initiates, maintains, and reinforces the anticipated behaviors. Extrinsic factors, such as financial incentives, have also often been portrayed in the literature as motivational factors, although Herzberg classified them as a hygiene factor that is associated only with job dissatisfaction (32). Behaviors refer to health service activities relating to the role of GPs in community detection and management of MCI.

The six building blocks in health system development recommended by the World Health Organization (WHO) (33) guided the determination of the sub-domains (second level) in relation to the “system platform that enables effective interactions between patients and care providers.” These sub-domains comprised: (i) leadership/governance; (ii) financing; (iii) workforce; (iv) service delivery; (v) information systems; and (vi) access to essential medicines. In our study, the assessment of leadership/governance covered both rules-based and outcome-based measurements. The former assessed regulatory and policy support to community detection and management of MCI, while the latter assessed practitioners' compliance with related rules. Financing referred to funding support to the health facilities that enabled adequate delivery of the required services and financial subsidies to the patients for use of the services. Health workforce engaging in community detection and management of MCI in the Chinese context included GPs, nurses, pharmacists, public health workers, and management and support staff in CHCs, and the hospital specialists to whom patients were referred. The delivery of community detection and management of MCI was designed as teamwork, involving both within- and across-organizational collaborations. The principles of patient-centered care apply, which emphasize effective communication, shared decision-making, mutual respect, and social support (34). Information systems were assumed to play an important role in facilitating the continuity and coordination of MCI care. Access to essential medicines was considered essential for managing risk factors associated with MCI (such as hypertension), despite a lack of effective medicines for treating MCI.

Subdividing the themes identified in the focus-group study resulted in a total of 44 items (third level): between two and eight items for each of the 15 sub-domains (Appendix 1).

#### Data collection

Data were collected from March 2020 to April 2020, involving two rounds of expert consultations. The consultations were conducted using an Excel spreadsheet containing the rating scales, which was distributed *via* email. Participants of these two rounds were the same individuals. They were given 2 weeks to complete the first round of consultations and 1 week to complete the second round of consultations. In addition to the consultation rating scales, socio-demographic data of the study participants were collected in the first round, including age (years), gender (male, female, others), years of work experience, area of expertise (general practice, community health service management, public health, neuropsychology), professional title (mid-career, associate professorial, professorial), and qualification (master's degree, doctorate degree). In the first round, participants were also asked to report their familiarity with the topic on a five-point Likert-type scale, ranging from unfamiliar to very familiar, as well as the judgement foundation (theoretical analysis, work experience, literature, and intuition) on which they made their ratings on the consultation scales.

Participants were asked to rate the importance of each of the domains, subdomains, and items on a five-point Likert-type scale: 5 = Essential, 4 = Important, 3 = Unsure, 2 = Unimportant, and 1 = Should not be included. An open text box was also attached to each scale, allowing the participants to provide comments and suggest changes. Two authors (YL and CL) reviewed the comments and suggested changes and revised the wording and categorization of the items for the consultation rating scales accordingly.

The revised consultation rating scales were used for the second round of expert consultations. Participants were provided with a summary of the results of the first-round consultations, including mean values for each item and explanations of any changes to rating scales between the rounds of consultation. They were again asked to rate the importance of each of the domains, sub-domains, and items on the same five-point Likert-type scale used in the first round, and an open text box was again provided. The participants were able to compare their responses with the first-round scores of the panel without knowing the identity of other panel members. They were allowed to either keep their original ratings or adjust their rating scores.

Data collection activities ended after the second round of consultations, as a high level of consensus was reached.

### Statistical analysis

Data were analyzed using Microsoft Excel.

Response rate [(Number of returned responses)/(Number of invitations)×100%] was calculated to reflect the enthusiasm of the eligible experts in participating in this study. Demographic characteristics of the participants were described using frequency distributions. The degree of authority of the consultation results was measured using the authority coefficient (Cr), which was determined by the reported familiarity (Cs) and the judgement foundation (Ca) of the participants:

1. Cr = (Cs + Ca)/2

In line with the literature (35–37), Cs scores were interpreted as: 0.9 = very familiar; 0.7 = familiar; 0.5 = somewhat familiar; 0.3 = a little familiar; 0.1 = unfamiliar. We used the scoring matrix that has been widely used in China and validated for Delphi studies on primary care services to calculate summed scores (36, 37): theoretical analysis (0.3 = a great deal; 0.2 = moderate; 0.1 = little); work experience (0.5 = a great deal; 0.4 = moderate; 0.3 = little); referring to literature (0.1 = a great deal; 0.1 = moderate; 0.1 = little); and intuition (0.1 = a great deal; 0.1 = moderate; 0.1 = little). A Cr value ≥ 0.7 was regarded as an indication of reasonable authority (36–38).

The degree of consensus of the participants was assessed using the coefficient of variation (CV) and the percentage of participant agreement with a rating of 4 (important) or 5 (essential). Mean and median scores were calculated to reflect the importance ratings. Retention of the domains, subdomains, and items required a median ≥ 4, mean ≥ 3.5, CV <0.25, and ≥ 80% agreement with a rating of 4 (important) or 5 (essential), in accordance with the literature (39–41).

## Results

### Socio-demographic characteristics of study participants

Eighty percent (24/30) of the invited experts participated in the first round of consultations. Two were not available after completing the first round of consultations, resulting in a reduction of the sample size to 22 for the second round. Over half of the participants were GPs and in the age group 30 to 39 years. The vast majority were women (>75%), resided in Shanghai (>87%), and had a master's degree qualification (>72%). Over two-thirds had worked for 10 to 19 years. About two-thirds of the participants had a senior professional title (Table 1).

**Table 1:** Characteristics of participants.

**Characteristics**	**Round one** **(*n* = 24)**		**Round two** **(*n* = 22)**	
	**Frequency**	**%**	**Frequency**	**%**
**Age (Years)**				
30–39	13	54.2	13	59.1
40–49	6	25.0	5	22.7
≥ 50	5	20.8	4	18.2
**Gender**				
Male	6	25.0	4	18.2
Female	18	75.0	18	81.8
**Work experience (Years)**				
10–19	16	66.7	16	72.7
20–29	5	20.8	4	18.2
≥ 30	3	12.5	2	9.1
**Speciality**				
General practice	13	54.2	12	54.5
Community service management	2	8.3	2	9.1
Public health	5	20.8	4	18.2
Neuropsychology	4	16.7	4	18.2
**Professional title**				
Mid-career professional	9	37.5	9	40.9
Associate professorial	8	33.3	7	31.8
Professorial	7	29.2	6	27.3
**Education degree**				
Master degree	18	75.0	16	72.7
Doctorate degree	6	25.0	6	27.3
**Work location**				
Shanghai	22	91.7	20	90.9
Beijing	2	8.3	2	9.1

### Consultation results

On average, the study participants had an authority coefficient (Cr) of 0.9 (SD = 0.1), with the smallest value exceeding the cut-off point of 0.7 (Appendix 2). Over 91% of study participants were very familiar or familiar with the study topic. About 71% made their ratings based on work experience to a great deal, compared with 42% reporting a moderate level of use of theoretical analysis. Intuition played a limited role in the expert judgement, with one-third reporting a moderate impact and two-thirds reporting little impact (Appendix 3).

The first round of consultations resulted in a removal of one sub-domain: “access to essential medicines.” Only 67% of participants rated it as important or essential, well below the cut-off point of 80%. The participants recommended the removal of this sub-domain simply because there are no effective medicines to treat MCI. The overall structure of the domains and sub-domains remained largely intact, despite the recommended re-naming of six sub-domains, removal of one sub-domain, and subdivision of two domains and two sub-domains. System support was divided into two: organizational effects and environmental support. “Healthcare setting-related factors” and “task-related factors” associated with patient engagement were moved and integrated into “organizational efforts.” More detailed descriptions regarding the sub-domains under “engaged patients” and “system support” were proposed by the experts and were adopted in the second round of consultations (Table 2).

**Table 2:** Delphi consultation rating scores on importance of measurement dimensions regarding community detection and management of mild cognitive impairment (MCI).

**Round one consultation (*n* = 24)**						**Round two consultation (*n* = 22)**					
**Dimension**	**Mean**	**Median**	**IQR**	**CV**	**Agreement (%)**	**Dimension**	**Mean**	**Median**	**IQR**	**CV**	**Agreement (%)**
**1. Prepared GPs**	4.9	5	[5, 5]	0.1	100.0	**1. Prepared GPs**	5.0	5	[5, 5]	0.0	100.0
1.1 Capacity	4.8	5	[5, 5]	0.1	100.0	1.1 Capacity	4.9	5	[5, 5]	0.1	100.0
1.2 Opportunity	4.6	5	[4, 5]	0.1	95.8	1.2 Opportunity	4.5	5	[4, 5]	0.2	95.6
1.3 Motivation	4.6	5	[5, 5]	0.2	91.7	1.3 Motivation	4.6	5	[4, 5]	0.2	90.9
1.4 Behavior	4.8	5	[5, 5]	0.1	100.0	1.4 Behavior	4.9	5	[5, 5]	0.2	100.0
**2. Engaged patients**	4.7	5	[4, 5]	0.1	100.0	**2. Engaged patients**	4.7	5	[4, 5]	0.1	100.0
2.1 Patient-related factors^♁^	4.8	5	[5, 5]	0.1	95.8	2.1 Patient attitudes toward MCI	4.8	5	[4, 5]	0.1	100.0
2.2 Disease-related factors^♁^	4.5	4	[4, 5]	0.1	95.8	2.2 Patient perceived barriers	4.9	5	[5, 5]	0.1	100.0
2.3 Doctor-related factors^♁^	4.3	4	[4, 5]	0.2	87.5	2.3 Doctors' encouragement	4.3	4	[4, 5]	0.2	90.9
2.4 Healthcare setting-related factors^−^	4.2	4	[4, 5]	0.2	87.5						
2.5 Task-related factors^−^	4.2	4	[4, 5]	0.2	87.5						
						2.4 Support of family caregivers^*^	4.6	5	[4, 5]	0.1	90.9
						2.5 Health literacy of patients^*^	4.3	4	[4, 5]	0.2	95.6
**3. System support^+^**	4.5	5	[4, 5]	0.2	95.8	**3. Organizational efforts** ^&^	4.6	5	[4, 5]	0.2	90.9
3.1 Information system^♁^	4.5	5	[4, 5]	0.2	87.5	3.1 Information support	4.7	5	[4, 5]	0.1	95.6
3.2 Management policy^♁^	4.8	5	[5, 5]	0.1	100.0	3.2 Management support	4.9	5	[5, 5]	0.1	100.0
3.3 Financial support^♁^	4.7	5	[4, 5]	0.1	95.8	3.3 Infrastructure support	4.9	5	[5, 5]	0.1	100.0
3.4 Teamwork	4.8	5	[5, 5]	0.1	100.0	3.4 Teamwork	4.6	5	[4, 5]	0.2	90.9
3.5 Essential medicine^X^	4.1	4	[3, 5]	0.2	66.7						
						**4. Environmental support** ^&^	4.7	5	[4, 5]	0.1	95.6
3.6 Service delivery^**+**^	4.6	5	[4, 5]	0.1	91.7	4.1 Health service arrangements**^&^**	4.8	5	[5, 5]	0.1	95.6
						4.2 Social environment**^&^**	4.2	4	[4, 5]	0.2	86.4

♁ Renamed;

+ Divided;

X Removed;

− Merged;

& Sub-divided domains or sub-domains;

* Added sub-domains.

IQR, Interquartile Range; CV, Coefficient of Variance.

The study participants recommended significant changes to the items in the first round of consultations, which included merging 11 items, dividing four items, removing two items, and adding 12 new items (Appendix 4). Eight items failed to reach the required 80% agreement on importance, three of which were removed as suggested by the participants. The removed items included two items measuring the removed sub-domain “access to essential medicine,” which also failed to meet the consensus criteria. Another removed item measured the “lack of control over non-pharmacological interventions by GPs” (63% agreement, 0.25 CV). Two items (“inconvenient location of health services” and “lack of testing/assessment facilities”) under the sub-domain “health care setting-related factors” were retained for the second round of consultations despite a lower than 80% agreement, since participants recommended re-categorizing and re-phrasing these items. Similarly, “peer pressure,” “perceived stigma,” and “impact on GPs from the task” were retained for the same reason (Table 3).

**Table 3:** Delphi consultation rating scores on importance of items regarding community detection and management of mild cognitive impairment (MCI).

**Round one consultation (*n* = 24)**						**Round two consultation (*n* = 22)**					
**Measurement Item**	**Mean**	**Median**	**IQR**	**CV**	**Agreement (%)**	**Measurement Item**	**Mean**	**Median**	**IQR**	**CV**	**Agreement (%)**
**1.1 GPs-Capacity**						**1.1 GPs-Capacity**					
1.1.1 Knowledge^♁^	4.8	5	[5, 5]	0.1	95.8	1.1.1 Knowledge of detecting MCI	4.9	5	[5, 5]	0.1	95.6
1.1.2 Skills^♁^	4.8	5	[5, 5]	0.1	100.0	1.1.2 MCI management skills	4.8	5	[5, 5]	0.1	100.0
1.1.3 Confidence **^+^**	4.2	4	[4, 5]	0.2	87.5	1.1.3 Assessing cognitive function**^&^**	4.6	5	[4, 5]	0.1	95.6
						1.1.4 Training certificate in MCI detection/management**^&^**	4.4	5	[4, 5]	0.2	86.4
						1.1.5 Communication with patients regarding MCI diagnosis**^&^**	4.5	5	[4, 5]	0.2	90.9
**1.2 GPs-Opportunity**						**1.2 GPs-Opportunity**					
1.2.1 MCI training^**#**^	4.7	5	[5, 5]	0.2	95.8						
1.2.2 Peer pressure^**#**^	3.9	4	[3, 5]	0.2	75.0						
1.2.3 Referral process**^−^**	4.6	5	[4, 5]	0.2	91.7						
1.2.4 Public health response^**#**^	4.7	5	[4, 5]	0.1	100.0						
						1.2.1 Patient cooperation^*^	4.7	5	[4, 5]	0.1	95.6
1.2.5 Caregivers‘ support**^−^**	4.7	5	[4, 5]	0.1	100.0						
1.2.6 Time allocation	4.3	4	[4, 5]	0.2	87.5	1.2.2 Time allocation	4.7	5	[4, 5]	0.1	100.0
1.2.7 Easily administered screening tools	4.8	5	[5, 5]	0.1	95.8	1.2.3 Easily administered screening tools	5.0	5	[5, 5]	0.0	100.0
1.2.8 Effective intervention methods	4.9	5	[5, 5]	0.1	100.0	1.2.4 Effective intervention methods	4.9	5	[5, 5]	0.1	100.0
**1.3 GPs-Motivation**						**1.3 GPs-Motivation**					
1.3.1 Belief in the value of MCI detection^♁^	4.5	5	[4, 5]	0.2	87.5	1.3.1 GPs‘ belief in the value of MCI detection and intervention	4.7	5	[4, 5]	0.1	90.9
1.3.2 Belief in the effectiveness of MCI intervention**^−^**	4.5	5	[4, 5]	0.2	91.7						
1.3.3 Role descriptions for GPs in MCI detection and management	4.5	5	[4, 5]	0.2	95.8	1.3.2 Responsibilities for GPs in MCI detection and management	4.6	5	[4, 5]	0.1	95.6
1.3.4 impact of the task on GPs^♁^	4.2	4	[4, 5]	0.2	79.2	1.3.3 Impacts on the career and income of GPs of performing the task	4.5	5	[4, 5]	0.2	86.4
**1.4 GPs-Behavior**						**1.4 GPs-Behavior**					
1.4.1 Disclosure^♁^	4.8	5	[4, 5]	0.1	100.0	1.4.1 Disclosure of suspected diagnosis	4.8	5	[4, 5]	0.1	100.0
1.4.2 Screening ^♁^	4.8	5	[5, 5]	0.1	95.8	1.4.2 Screening suspected patients	5.0	5	[5, 5]	0.0	100.0
1.4.3 Referral^♁^	4.7	5	[4, 5]	0.1	95.8	1.4.3 Referral to a specialist	4.7	5	[4, 5]	0.1	100.0
1.4.4 Treatment^♁^	4.3	5	[4, 5]	0.2	83.3	1.4.4 MCI prevention and management	4.6	5	[4, 5]	0.1	100.0
						1.4.5 Patient follow-up^*^	4.7	5	[4, 5]	0.1	100.0
**2.1 Patient-related factors**						**2.1 Patient attitudes toward MCI**					
2.1.1 Patient awareness of cognitive disorder**^+^**	4.8	5	[4, 5]	0.1	100.0	2.1.1 Patient perceived susceptibility to MCI**^&^**	4.9	5	[5, 5]	0.1	100.0
						2.1.2 Patient perceived seriousness of MCI**^&^**	4.9	5	[5,5]	0.1	100.0
2.1.2 Perceived stigma^**#**^	4.3	5	[4, 5]	0.2	79.2						
**2.2 Disease-related factors**						**2.2 Patient perceived barriers**					
2.2.1 Limited effects on daily activity**^−^**	4.3	4	[4, 5]	0.2	83.3						
2.2.2 Late presentation of symptoms**^−^**	4.4	5	[4, 5]	0.2	87.5						
2.2.3 No equipment/laboratory tools to confirm diagnosis**^−^**	4.4	5	[4, 5]	0.2	91.7						
2.2.4 No effective medicine^♁^	4.3	4	[4, 5]	0.2	91.7	2.2.1 Difficulties to engage in non-pharmaceutical interventions	4.7	5	[4, 5]	0.1	95.6
						2.2.2 Perceived stigma	4.4	4	[4, 5]	0.1	95.6
						2.2.3 Financial concerns^*^	4.4	5	[5, 5]	0.2	90.9
**2.3 Doctor-related factors**						**2.3 Doctors' encouragement**					
2.3.1 Doctor-patient relationship	4.5	5	[4, 5]	0.1	95.8	2.3.1 Doctor-patient relationship	4.5	5	[4, 5]	0.2	90.9
2.3.2 Patient trust in GPs for handling MCI	4.3	4	[4, 5]	0.2	91.7	2.3.2 Patient trust in GPs for handling MCI	4.5	5	[4, 5]	0.1	95.6
						2.3.3 Attention to MCI demonstrated by GPs^*^	4.6	5	[4, 5]	0.1	86.4
**2.4 Health care setting-related factors**						**2.4 Support of family caregivers**					
2.4.1 Inconvenient location of health services**^−^**	3.8	4	[3, 5]	0.2	66.7						
2.4.2 Lack of testing/assessment facilities ^**#**^	4.0	4	[3, 5]	0.2	66.7						
						2.4.1 MCI knowledge of caregivers^*^	4.5	5	[4, 5]	0.1	90.9
						2.4.2 Perceived role of caregivers in MCI detection and management^*^	4.4	4	[4, 5]	0.2	90.9
						2.4.3 Support of caregivers to MCI detection and management^*^	4.6	5	[4, 5]	0.1	100.0
						2.4.4 Family relationship ^*^	4.5	5	[4, 5]	0.1	95.6
**2.5 Task-related factors**						**2.5 Health literacy of patients**					
2.5.1 Time-consuming process in MCI detection and management**^−^**	4.5	5	[4, 5]	0.1	95.8						
2.5.2 Lack of control of GPs over non-pharmacological interventions**^X^**	3.7	4	[3, 5]	0.3	62.5						
						2.5.1 Health knowledge of patients^*^	4.3	5	[4, 5]	0.2	90.9
						2.5.2 Patient self-assessment of health^*^	4.3	5	[5, 5]	0.2	90.9
						2.5.3 Financial and living conditions of patients^*^	4.5	5	[4, 5]	0.2	95.6
**3.1 Information system**						**3.1 Information system**					
3.1.1 Screening alert system^♁^	4.4	5	[4, 5]	0.2	87.5	3.1.1 Alert for screening and follow-up	4.6	5	[4, 5]	0.1	95.6
3.1.2 Follow-up system**^−^**	4.5	4	[4, 5]	0.1	100.0						
3.1.3 Electronic screening scale**^+^**	4.7	5	[4, 5]	0.1	100.0	3.1.2 Electronic screening scale**^&^**	4.7	5	[4, 5]	0.1	100.0
						3.1.3 Electronic referral request**^&^**	4.6	5	[4, 5]	0.1	100.0
**3.2 Management policy**						**3.2 Management support**					
3.2.1 Incorporating MCI detection and management into daily practice^♁^	4.7	5	[4, 5]	0.1	95.8	3.2.1 Convenient service procedures in MCI detection and management	4.9	5	[5, 5]	0.1	100.0
3.2.2 Evaluation of intervention performance^♁^	4.4	4	[4, 5]	0.1	95.8	3.2.2 Mechanisms in place for service performance assessment	4.6	5	[4, 5]	0.1	100.0
**3.3 Financial support**						**3.3 Infrastructure support**					
3.3.1 Investment in infrastructure**^+^**	4.6	5	[4, 5]	0.1	100.0	3.3.1 Establishment of memory clinic**^&^**	4.8	5	[5, 5]	0.1	100.0
						3.3.2 Investment in screening/diagnostic facilities	4.7	5	[4, 5]	0.1	90.9
						3.3.3 Investment in intervention facilities**^&^**	4.7	5	[4, 5]	0.1	100.0
3.3.2 Pay for performance**^−^**	4.6	5	[4, 5]	0.1	91.7						
**3.4 Teamwork**						**3.4 Teamwork**					
3.4.1 Coordinated teamwork^♁^	4.5	5	[4, 5]	0.2	91.7	3.4.1 Establishment of effective teamwork with accountability	4.6	5	[4, 5]	0.1	95.6
3.4.2 Responsibility of team members**^−^**	4.7	5	[4, 5]	0.1	95.8						
						3.4.2 MCI training relating to different roles	4.7	5	[4, 5]	0.1	100.0
						3.4.3 Collegial (peer) push	4	4	[4, 4]	0.2	90.9
**3.5 Essential medicine**											
3.5.1 Provision of essential medicine**^X^**	4.3	4	[4, 5]	0.2	79.2						
3.5.2 Cost-effectiveness of essential medicine**^X^**	4.2	4	[3, 5]	0.2	75.0						
**3.6 Service delivery**											
3.6.1 Inclusion of MCI in primary care service package^**#**^	4.8	5	[5, 5]	0.1	95.8						
3.6.2 Support from the community^**#**^	4.5	5	[4,5]	0.2	100.0						
						**4.1 Health service arrangements**					
						4.1.1 Inclusion of MCI in primary care service package	5	5	[5, 5]	0.1	95.6
						4.1.2 Inclusion of MCI screening in routine health check-up list for old adults^*^	5	5	[8, 5]	0.1	95.6
						**4.2 Social environments**					
						4.2.1 Public awareness of MCI	5	5	[4, 5]	0.1	95.6
						4.2.2 Community involvement	5	5	[5, 5]	0.1	100.0

♁ Renamed;

+ Divided;

X Removed;

− Merged;

& Sub-divided domains or sub-domains;

* Added sub-domains.

IQR, Interquartile Range. CV, Coefficient of Variance.

Twelve items were added for the second-round consultations following the recommendations of the study participants. These included “patient cooperation,” measuring opportunity for GPs; “patient follow-up,” measuring GPs' behavior; “financial concerns” and “attention to MCI demonstrated by GPs,” measuring “patient perceived barriers”; and “inclusion of MCI screening in a routine health check-up list for older adults,” measuring “health service arrangements.” In addition, four items were added to measure the added sub-domain “support of family caregivers,” and three items were added to measure the added sub-domain “patient health literacy” (Table 3).

Four items were suggested by the study participants to be sub-divided using more specific descriptions. “Confidence” of GPs in detecting and managing MCI was divided into three aspects: cognitive function assessment, training certificate in MCI detection/management, and communication with patients regarding the MCI diagnosis. “Patient awareness of cognitive disorder” was divided into two: “patient perceived susceptibility to MCI” and “patient perceived seriousness of MCI.” “Electronic screening scale” was divided into “electronic screening scale” and “electronic referral request.” “Investment in infrastructure” was divided into “establishment of a memory clinic” and “investment in intervention facilities” in addition to “investment in screening/diagnostic facilities.”

Eleven items were merged with others because of their inherent links. For example, two duplicated items measuring “caregivers' support” under “opportunity of GPs” and “engaged patients,” respectively, were merged. Belief in the value of MCI detection and the effectiveness of MCI intervention were pulled together. “Information alert system” covered both “follow-up alert” and “screening alert.” Individual responsibility and coordination were deemed equally important as an indication of effective teamwork. The three items measuring “disease-related factors” were amalgamated into the sub-domain “patient attitudes toward MCI.” All matters relating to service procedures (e.g., location, time, referral, and rewards) were amalgamated into the sub-domain “management support.”

**Figure 1:**
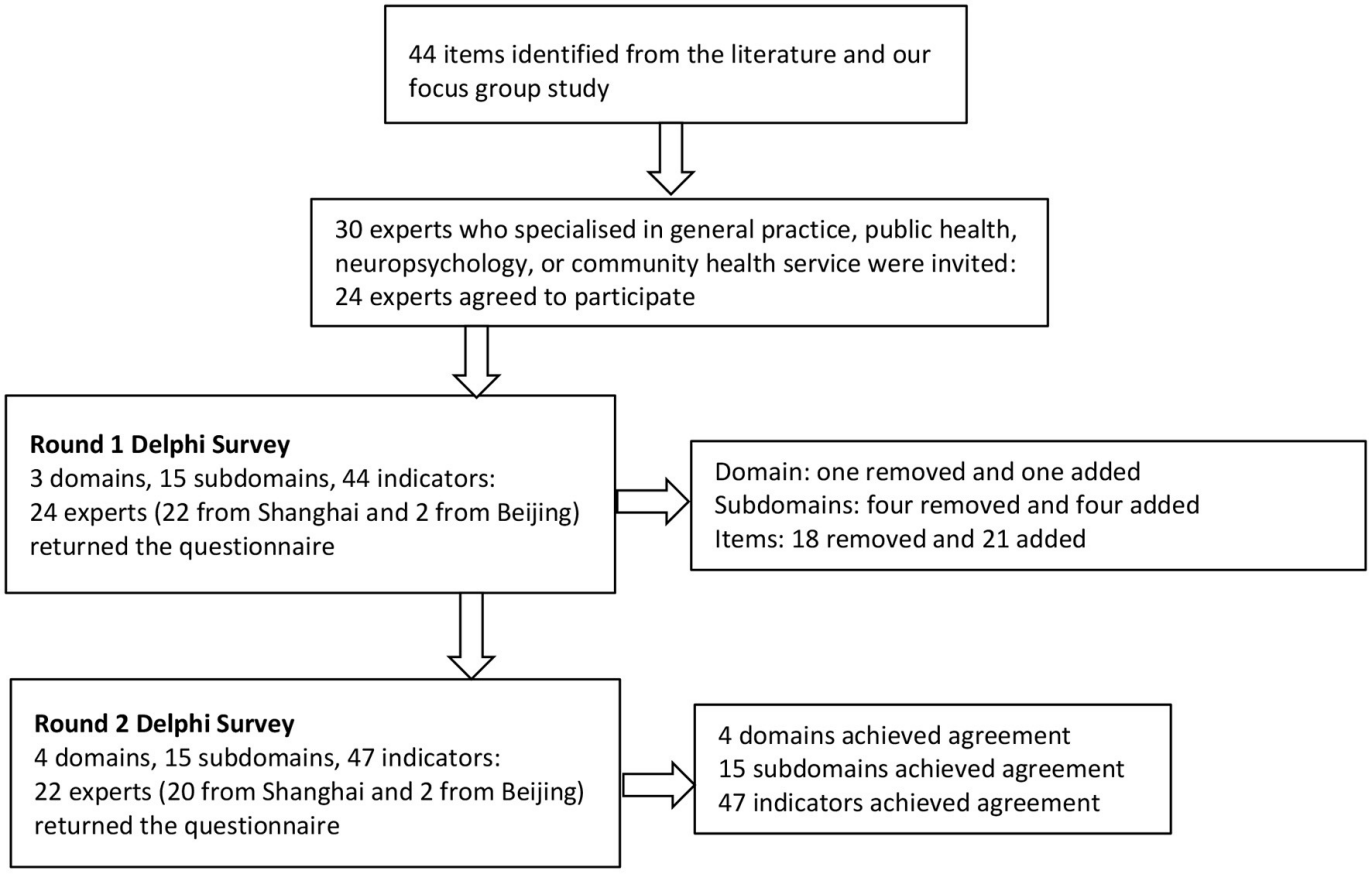
Diagram of the Delphi process.

Almost all items were rephrased using more specific descriptions after the first-round consultations. The second-round consultation rating scales contained 4 domains, 15 sub-domains, and 47 items, all of which met the consensus criteria (Table 3). No further changes were recommended by the study participants. Figure 1 summarizes the entire Delphi process.

## Discussion

The current study applied the Delphi consultation method to establish expert consensus on the conditions required for successful MCI detection and management in primary care in China. Consensus was reached among the study participants of our study on 47 conditions relating to prepared GPs (17 items), engaged patients (15 items), organizational efforts (11 items), and environmental support (4 items). The experts endorsed the CCM, although they suggested minor modifications to the CCM by dividing system support into two: those factors overseen by the organization and those imposed by the broad environment. Organizational efforts are necessary to ensure well-coordinated teamwork with adequate infrastructure support. However, organizational efforts alone are not enough, given that the behaviors of both patients and care providers are heavily influenced by the environments in which they live and work.

### Prepared GPs

GPs' capacity (5 items), opportunity (4 items), motivation (3 items), and behavior (5 items) were all deemed relevant or important by the expert panel of our study for MCI detection and management. Knowledge and skill acquisition are always the first step to building capacity for new medical services (42). The study participants agreed that GPs need to feel confident about cognitive function assessment and communication (disclosure) with patients. Confidence was considered to be associated with having completed a training certificate in MCI detection/management. These results are consistent with the findings of previous studies. A report from a global panel of clinicians and cognitive neuroscientists on current challenges that hamper widespread cognitive performance assessment has shown that GPs may struggle with choosing appropriate tools for assessing cognitive function (43). It is not uncommon for primary care providers to feel a lack of confidence in disclosing MCI diagnosis to patients for a variety of reasons. For example, GPs may fear that disclosing a diagnosis of cognitive impairment might damage their relationship with patients because of the potential negative impacts on the patient receiving the diagnostic information (44).

Apart from the availability of technologies and time, the expert panel suggested “patient cooperation” may provide opportunity for GPs to conduct MCI detection and management. Empirical evidence from previous studies has shown that high levels of patient cooperation are indeed associated with improved health outcomes, enhanced patient satisfaction, and better adherence to treatment (34, 45).

The expert panel agreed that belief in the value of MCI care, a sense of responsibility, and rewards such as career advancement and remuneration are motivators of GPs to conduct detection and management of MCI. These factors align well with a contemporary motivation theory. In a recent systematic review, belief in the value of work and responsibility were labeled as intrinsic motivation factors, while income remuneration and career advancement were considered extrinsic driving factors (46).

The expert panel agreed that GPs can provide a range of MCI-related services, including screening, referral, communication, intervention, and follow-up evaluation. According to a global working group comprising international experts on MCI and Alzheimer's disease, GPs should coordinate post-diagnostic interventions and track the progression of MCI to early Alzheimer's disease (43).

### Engaged patients

The expert panel agreed that patient engagement is associated with patient attitudes (2 items), perceived barriers (3 items), health literacy (3 items), doctors' encouragement (3 items), and the support of family caregivers (4 items). These results are supported by evidence derived from other studies. The HBM proposes that the health actions of patients are triggered by perceived susceptibility to the disease and the seriousness of the condition (42). Unfortunately, memory loss has commonly been considered as part of normal aging among people living with MCI (10, 47), leading to inaction.

Our study identified major patient-related barriers to MCI care, including difficulties in engaging patients in non-pharmacological interventions, social stigma, and financial concerns. Indeed, MCI interventions may not take effect until after 3 months of intervention, according to a systematic review (48). Patients may find it difficult to comply with long-term MCI interventions (49). Adding to the complexity of achieving effective patient engagement is the social stigma attached to MCI, which can lead to internalized shame or social isolation (50). In a health system yet to achieve universal health coverage, such as in China (51), it is understandable that “financial concerns” can also become a hurdle for accessing MCI-related services. Some therapies such as cognitive intervention therapies are not covered by social health insurance programs in China.

The expert panel agreed that patients may engage in MCI detection and management to various degrees, depending on their health knowledge, self-assessed health, and financial and living conditions. These results align with the definition of health literacy from a systematic review (52): “Health literacy is the ability of an individual to obtain and translate knowledge and information in order to maintain and improve health in a way that is appropriate to the individual and system contexts.”

The role doctors play in encouraging patients to engage in MCI detection and management activities is shaped by the individual commitment of the doctor, the doctor-patient relationship, and patient trust in the doctor, according to the expert consensus found in our study. Widespread distrust and poor doctor-patient relationships have recently attracted serious concern in China (18). However, according to a qualitative study in the United Kingdom, even if patients place high trust in their doctors, they may still dismiss concerns about MCI (53).

The expert panel agreed that family caregivers also play a role in patient engagement, which is associated with their knowledge about MCI, family relationships, self-perception of their role as caregivers, and the support available to them. Empirical evidence from the United States shows that a lack of knowledge and coping strategies to support people with MCI are common in family caregivers (54), although good family relationships can encourage patients to engage in daily activities that are beneficial to MCI (55). A systematic review found that family caregivers tend to be reluctant to acknowledge their responsibilities relating to MCI care due to predictable “role strains” (56). As a result, family caregivers may need some additional support (57).

### Organizational efforts

The current study's expert panel categorized organizational efforts into information support (3 items), infrastructure support (3 items), management support (2 items), and teamwork (3 items). These are closely aligned with China's efforts in strengthening community management of chronic diseases, such as hypertension and diabetes (14). Indeed, information sharing is essential to ensure continuity and coordination of care, which is critical for improving patient safety and patient care outcomes, especially for those with chronic diseases (18).

The expert panel endorsed memory clinics as needed infrastructure for MCI detection and management, in addition to investment in screening instruments and intervention facilities. A study in China shows that the number of memory clinics in tertiary hospitals is considered by health professionals to be inadequate, and the situation is much worse in primary care settings (58). Empirical evidence from some high-income countries indicates that memory clinics can provide primary care providers with accessible, efficient, and cost-effective tools to handle memory problems (59).

Management support (in terms of service procedure and performance assessment) is needed to incentivise primary care workers to commit to MCI-related services, according to our expert panel. This perhaps reflects the broad management culture in China. Pay for performance based on the volume of services provided has been widely adopted as an instrument to incentivise health workers (60), despite criticism about the potential for perverse incentives (61).

GP-led teamwork has been a predominant model in community health services in China (13), which was also a necessary condition for managing MCI endorsed by our expert. The provision of “essential medicines,” however, was deemed irrelevant to MCI management by the expert panel. In addition to the lack of effective medicines to treat MCI (62), medicines listed in China's essential medicines list have been widely available and affordable from a variety of health facilities (14). This may have led the experts to believe that essential medicines are irrelevant to MCI.

### Environmental support

The expert panel of our study was concerned about not only the health service arrangements in place for detection and management of MCI (2 items) but also the broad social environment in which health services occur (2 items). They endorsed the call to include MCI- and dementia-related services into the national package of essential public health services (63). Unfortunately, the current package promulgated in 2017 covers only hypertension, diabetes, psychosis, and tuberculosis (64). Many CHCs are providing regular health check-ups for older people free of charge. The expert panel agreed that MCI screening should be included in the check-up list. This result aligns well with the recommendations of the report from the organization Alzheimer's Disease China (ADC) (65).

Public awareness of MCI and community involvement in MCI detection and management were endorsed by the expert panel of our study for measuring the supportiveness of the social environment in detecting and managing MCI. Establishing a “friendly community” has been widely accepted internationally as a strategy to enable people with cognitive impairment to feel supported and be integrated into their local communities (66). High public awareness and social support can also be used in fighting the social stigma attached to MCI (50).

## Strengths and limitations

The Delphi consultations adopted in our study were built on robust theoretical frameworks and involved experts with a high level of authority. Members of the expert panel made their judgements mainly based on work experience and theoretical analyses. However, expert consensus is considered the lowest level of evidence in evidence-based medicine (67). A limitation of the method is that most of the panel members were located in Shanghai, due to a lack of community MCI detection and management programs in other regions; neither did the panel involve MCI patients and caregivers, who could have provided an alternative perspective. The sample size of the current study is adequate, but relatively small. Although there may be many GPs and others with experience of dealing with MCI in China, we were not able to increase the sample size due to time and resource restrictions and the difficulty of identifying potential additional participants. Instead, we subsequently conducted a large questionnaire survey of GPs regarding their knowledge of, and attitudes and behaviors toward, MCI-related services.

## Conclusion

There is expert consensus about the applicability of CCM in MCI-related service arrangements. Successful detection and management of MCI in primary care in China requires a dedicated and competent workforce of general practitioners, as well as engagement of patients and family caregivers. Adequate support from healthcare organizations, health system arrangements, and the broader society is needed to facilitate effective interactions between GPs and patients and efficient delivery of the services.

Our study developed a checklist of 47 necessary conditions required for successfully detecting and managing MCI in primary care in China. This checklist has the potential to serve as a framework for assessing MCI programs, and help policymakers and health service managers to nurture a supportive environment that enables efficient and effective service delivery relating to MCI in primary care. Further studies are needed to validate the checklist for the above-mentioned purposes. Adaptations of the checklist will likely be required depending on the context in which it is used.

## Data availability statement

The raw data supporting the conclusions of this article will be made available by the authors, without undue reservation.

## Ethics statement

The studies involving human participants were reviewed and approved by the Human Ethics Committee of La Trobe University (HEC20125) and the Medical Ethics Committee of Yangpu Hospital, Shanghai, China (LL-2019-SCI-004). Written informed consent for participation was not required for this study in accordance with the national legislation and the institutional requirements.

## Author contributions

YL and CL contributed to the conceptualization and design of this study, analyzed, and interpreted the data. DY provided support to data collection. The manuscript was drafted by YL and revised by CL and YW. The final version was reviewed and approved by all authors.

## Funding

This project was supported by the Australian Government Research Training Program Fees Offset (RTP Fees Offset) and the La Trobe University Full Fee Research Scholarship (LTUFFRS). The research was partly funded by Shanghai Municipal Health Commission, China (201940495). The funding bodies have not had any involvement in the design, execution, or reporting of the study.

## Conflict of interest

The authors declare that the research was conducted in the absence of any commercial or financial relationships that could be construed as a potential conflict of interest.

## Publisher's note

All claims expressed in this article are solely those of the authors and do not necessarily represent those of their affiliated organizations, or those of the publisher, the editors and the reviewers. Any product that may be evaluated in this article, or claim that may be made by its manufacturer, is not guaranteed or endorsed by the publisher.
