# Supplementary material for: Conditions required to ensure successful detection and management of mild cognitive impairment in primary care: A Delphi consultation study in China
Source: Front Public Health. 2022 Sep 23;10:943964. doi: 10.3389/fpubh.2022.943964 (PMC9540221; doi:10.3389/fpubh.2022.943964)
Supplement: Supplementary file 1 [file Table_1.pdf]

Appendix 1 Delphi questionnaire

Section I Personal Information

1. Gender

☐ Male
☐ Female
☐ Others

2. Age (years)

☐ < 30
☐ 30-39
☐ 40-49
☐ ≥50

3. Educational qualification

☐ Associate degree
☐ Bachelor degree
☐ Master degree
☐ Doctorate degree

4. Professional title

☐ Primary
☐ Mid-career
☐ Associate professorial
☐ Professorial

5. Speciality

☐ General practice
☐ Public health
☐ Community health service management
☐ Neuropsychology
☐ Others

6. Work experience (years)

☐ < 10
☐ 10-19
☐ 20-29
☐ ≥30

Section II Conditions for successful detection and management of mild cognitive impairment (MCI) in primary care

【Instructions】

1. We drafted a three-level indicator system for successful detection and management of MCI in primary care, comprising 3 domains (first level), 15 subdomains (second level), and 44 items (third level).
2. Please rate the importance of each domain, subdomain, and item based on your professional judgment. Importance refers to the contribution of each item, subdomain, and domain for successful detection and management of MCI in primary care. Each is rated on a five-point Likert-type scale ranging from 1 (least important) to 5 (essential). You are invited to provide comments and suggest changes in an open text box attached to each Likert-type scale.

Table 1. Your familiarity with the survey content

	**Familiarity with the survey content**				
	**Very familiar (5)**	**Familiar (4)**	**Somewhat familiar (3)**	**A little familiar (2)**	**Unfamiliar (1)**
Self-rating	☐	☐	☐	☐	☐

Table 2. The foundation of your judgement and the extent of its impact

**Judgment foundation**	**Impact**		
	**Great (3)**	**Medium (2)**	**Little (1)**
1. Theoretical analysis	☐	☐	☐
2. Practical experience	☐	☐	☐
3. Referring to literature	☐	☐	☐
4. Intuitive perception	☐	☐	☐

Table 3. Rating scale on the importance of domains

**Domains**	**Essential (5)**	**Important (4)**	**Unsure (3)**	**Unimportant (2)**	**Least important (1)**	**Should not be included**	**Suggested changes**
1. Prepared GPs	☐	☐	☐	☐	☐		
2. Engaged patients	☐	☐	☐	☐	☐		
3. System support	☐	☐	☐	☐	☐		
Suggested domains							

Table 4. Rating scale on the importance of subdomains

Domains	Subdomains	Essential (5)	Important (4)	Unsure (3)	Unimportant (2)	Least important (1)	Should not be included	Suggested changes
1. Prepared GPs	1.1 Capacity to detect and manage MCI	☐	☐	☐	☐	☐		
	1.2 Opportunity to detect and manage MCI	☐	☐	☐	☐	☐		
	1.3 Motivation to detect and manage MCI	☐	☐	☐	☐	☐		
	1.4 Behaviour to detect and manage MCI	☐	☐	☐	☐	☐		
	Suggested subdomains							
2. Engaged patients	2.1 Patient-related factors	☐	☐	☐	☐	☐		
	2.2 Disease-related factors	☐	☐	☐	☐	☐		
	2.3 Doctor-related factors	☐	☐	☐	☐	☐		
	2.4 Healthcare setting-related factors	☐	☐	☐	☐	☐		
	2.5 Task-related factors	☐	☐	☐	☐	☐		
	Suggested subdomains							
3. System support	3.1 Information system	☐	☐	☐	☐	☐		
	3.2 Management policy	☐	☐	☐	☐	☐		
	3.3 Financial support	☐	☐	☐	☐	☐		
	3.4 Teamwork	☐	☐	☐	☐	☐		
	3.5 Essential medicine	☐	☐	☐	☐	☐		
	3.6 Service delivery	☐	☐	☐	☐	☐		
	Suggested subdomains							

Table 5. Rating scale on the importance of items

Domains and subdomains	Items	Essential (5)	Important (4)	Unsure (3)	Unimportant (2)	Least important (1)	Should not be included	Suggested changes
1. Prepared GPs								
1.1 Capacity to detect and manage MCI	1.1.1 MCI related Knowledge	☐	☐	☐	☐	☐		
	1.1.2 MCI related skills	☐	☐	☐	☐	☐		
	1.1.3 Confidence to detect and mange MCI	☐	☐	☐	☐	☐		
	Suggested indicators							
1.2 Opportunity to detect and manage MCI	1.2.1 MCI training	☐	☐	☐	☐	☐		
	1.2.2 Peer pressure	☐	☐	☐	☐	☐		
	1.2.3 Referral process	☐	☐	☐	☐	☐		
	1.2.4 Public health response	☐	☐	☐	☐	☐		
	1.2.5 Caregivers' support	☐	☐	☐	☐	☐		
	1.2.6 Time allocation	☐	☐	☐	☐	☐		
	1.2.7 Easily administered screening tools	☐	☐	☐	☐	☐		
	1.2.8 Effective intervention methods	☐	☐	☐	☐	☐		
	Suggested indicators							
1.3 Motivation to detect and manage MCI	1.3.1 Belief in the value of MCI detection	☐	☐	☐	☐	☐		
	1.3.2 Belief in the effectiveness of MCI intervention	☐	☐	☐	☐	☐		
	1.3.3 Role descriptions for GPs in MCI detection and management	☐	☐	☐	☐	☐		
	1.3.4 Impact of the task on GPs	☐	☐	☐	☐	☐		
	Suggested indicators							
1.4 Behaviour to detect and manage MCI	1.4.1 Disclosure of MCI diagnosis	☐	☐	☐	☐	☐		
	1.4.2 MCI Screening	☐	☐	☐	☐	☐		
	1.4.3 MCI patients` referral	☐	☐	☐	☐	☐		
	1.4.4 MCI treatment	☐	☐	☐	☐	☐		
	Suggested indicators							
**2.** Engaged patients								
2.1 Patient-related factors	2.1.1 Patient awareness of cognitive disorder	☐	☐	☐	☐	☐		
	2.1.2 Perceived stigma	☐	☐	☐	☐	☐		
	Suggested indicators							
2.2 Disease-related factors	2.2.1 Limited effects on daily activity	☐	☐	☐	☐	☐		
	2.2.2 Late presentation of symptoms	☐	☐	☐	☐	☐		
	2.2.3 Equipment or laboratory tools to confirm diagnosis	☐	☐	☐	☐	☐		
	2.2.4 No effective medicine	☐	☐	☐	☐	☐		
	Suggested indicators							
2.3 Doctor-related factors	2.3.1 Doctor-patient relationship	☐	☐	☐	☐	☐		
	2.3.2 Patient trust in GPs for handling MCI	☐	☐	☐	☐	☐		
	Suggested indicators							
2.4 Healthcare setting-related factors	2.4.1 Inconvenient location of health services	☐	☐	☐	☐	☐		
	2.4.2 Lack of testing/assessment facilities	☐	☐	☐	☐	☐		
	Suggested indicators							
2.5 Task-related factors	2.5.1 Time-consuming process in MCI detection and management	☐	☐	☐	☐	☐		

	2.5.2 Lack of control of GPs over non-pharmacological interventions	☐	☐	☐	☐	☐		
	Suggested indicators							
3. System support								
3.1 Information system	3.1.1 Screening alert system	☐	☐	☐	☐	☐		
	3.1.2 Follow-up system	☐	☐	☐	☐	☐		
	3.1.3 Electronic screening scale	☐	☐	☐	☐	☐		
	Suggested indicators							
3.2 Management policy	3.2.1 Incorporating MCI detection and management into daily practice	☐	☐	☐	☐	☐		
	3.2.2 Evaluation of intervention performance	☐	☐	☐	☐	☐		
	Suggested indicators							
3.3 Financial support	3.3.1 Investment in infrastructure	☐	☐	☐	☐	☐		
	3.3.2 Pay for performance	☐	☐	☐	☐	☐		
	Suggested indicators							
3.4 Teamwork	3.4.1 Coordinated teamwork	☐	☐	☐	☐	☐		
	3.4.2 Responsibility of team members	☐	☐	☐	☐	☐		
	Suggested indicators							
3.5 Essential medicine	3.5.1 Provision of essential medicine	☐	☐	☐	☐	☐		
	3.5.2 Cost-effectiveness of essential medicine	☐	☐	☐	☐	☐		
	Suggested indicators							
3.6 Service delivery	3.6.1 Inclusion of MCI in primary care service package	☐	☐	☐	☐	☐		
	3.6.2 Support from the community	☐	☐	☐	☐	☐		
	Suggested indicators							

Thanks for your participation！
