# Supplementary material for: Conditions required to ensure successful detection and management of mild cognitive impairment in primary care: A Delphi consultation study in China
Source: Front Public Health. 2022 Sep 23;10:943964. doi: 10.3389/fpubh.2022.943964 (PMC9540221; doi:10.3389/fpubh.2022.943964)
Supplement: Supplementary file 2 [file Table_2.DOCX]

| **Expert** | **Judgement foundation*** | | | | | **Reported familiarity (Cs)** | **Authority coefficient (Cr)** |
| --- | --- | --- | --- | --- | --- | --- | --- |
|  | **Theoretical analysis** | **Practical experience** | **Referring to literature** | **Intuitive perception** | **Ca** |  |  |
| 1 | 0.3 | 0.5 | 0.1 | 0.1 | 1.0 | 0.9 | 0.95 |
| 2 | 0.3 | 0.4 | 0.1 | 0.1 | 0.9 | 0.7 | 0.8 |
| 3 | 0.2 | 0.5 | 0.1 | 0.1 | 0.9 | 0.9 | 0.9 |
| 4 | 0.3 | 0.4 | 0.1 | 0.1 | 0.9 | 0.9 | 0.9 |
| 5 | 0.3 | 0.5 | 0.1 | 0.1 | 1.0 | 0.7 | 0.85 |
| 6 | 0.3 | 0.5 | 0.1 | 0.1 | 1.0 | 0.9 | 0.95 |
| 7 | 0.3 | 0.5 | 0.1 | 0.1 | 1.0 | 0.9 | 0.95 |
| 8 | 0.3 | 0.5 | 0.1 | 0.1 | 1.0 | 0.9 | 0.95 |
| 9 | 0.3 | 0.4 | 0.1 | 0.1 | 0.9 | 0.9 | 0.9 |
| 10 | 0.3 | 0.4 | 0.1 | 0.1 | 0.9 | 0.9 | 0.9 |
| 11 | 0.2 | 0.5 | 0.1 | 0.1 | 0.9 | 0.7 | 0.8 |
| 12 | 0.3 | 0.4 | 0.1 | 0.1 | 0.9 | 0.9 | 0.9 |
| 13 | 0.2 | 0.5 | 0.1 | 0.1 | 0.9 | 0.9 | 0.9 |
| 14 | 0.3 | 0.5 | 0.1 | 0.1 | 1.0 | 0.5 | 0.75 |
| 15 | 0.3 | 0.4 | 0.1 | 0.1 | 0.9 | 0.9 | 0.9 |
| 16 | 0.3 | 0.5 | 0.1 | 0.1 | 1.0 | 0.9 | 0.95 |
| 17 | 0.2 | 0.5 | 0.1 | 0.1 | 0.9 | 0.9 | 0.9 |
| 18 | 0.2 | 0.5 | 0.1 | 0.1 | 0.9 | 0.9 | 0.9 |
| 19 | 0.3 | 0.5 | 0.1 | 0.1 | 1.0 | 0.5 | 0.75 |
| 20 | 0.2 | 0.5 | 0.1 | 0.1 | 0.9 | 0.9 | 0.9 |
| 21 | 0.2 | 0.5 | 0.1 | 0.1 | 0.9 | 0.9 | 0.9 |
| 22 | 0.2 | 0.5 | 0.1 | 0.1 | 0.9 | 0.9 | 0.9 |
| 23 | 0.2 | 0.4 | 0.1 | 0.1 | 0.8 | 0.9 | 0.85 |
| 24 | 0.2 | 0.5 | 0.1 | 0.1 | 0.9 | 0.9 | 0.9 |
| * Scoring algorithm for judgement foundation   \| Judgment foundation \| Impact \| \| \| \| --- \| --- \| --- \| --- \| \| Great \| Medium \| Little \| \| Theoretical analysis \| 0.3 \| 0.2 \| 0.1 \| \| Work experience \| 0.5 \| 0.4 \| 0.3 \| \| Referring to literature \| 0.1 \| 0.1 \| 0.1 \| \| Intuitive perception \| 0.1 \| 0.1 \| 0.1 \| \| Source: Wang YY. Developing a Patient Safety Culture Assessment Scale for Maternal and Child Health Institutions. Beijing: Peking University; 2017. Retrieved from http://ir.bjmu.edu.cn/handle/400002259/148313 \| \| \| \| | | | | | | | |

**Appendix 2 Characteristics of authority coefficient (N=24)**
