# Supplementary material for: Conditions required to ensure successful detection and management of mild cognitive impairment in primary care: A Delphi consultation study in China
Source: Front Public Health. 2022 Sep 23;10:943964. doi: 10.3389/fpubh.2022.943964 (PMC9540221; doi:10.3389/fpubh.2022.943964)
Supplement: Supplementary file 3 [file Table_3.DOCX]

| Judgement Sources | Great | |  | Medium | |  | Little | |
| --- | --- | --- | --- | --- | --- | --- | --- | --- |
|  | Frequency | Percentage |  | Frequency | Percentage |  | Frequency | Percentage |
| Theoretical Analysis | 14 | 58.3 |  | 10 | 41.7 |  |  |  |
| Practical Experience | 17 | 70.8 |  | 7 | 29.2 |  |  |  |
| Referring to Literature | 15 | 62.5 |  | 9 | 37.5 |  |  |  |
| Intuitive perception |  |  |  | 8 | 33.3 |  | 16 | 66.7 |

**Appendix 3. Frequency distributions of expert judgement sources (N=24)**
