# Supplementary material for: Conditions required to ensure successful detection and management of mild cognitive impairment in primary care: A Delphi consultation study in China
Source: Front Public Health. 2022 Sep 23;10:943964. doi: 10.3389/fpubh.2022.943964 (PMC9540221; doi:10.3389/fpubh.2022.943964)
Supplement: Supplementary file 4 [file Table_4.DOCX]

**Appendix 4 Distribution of indicators of two-rounds Delphi survey by agreement**

| Items | Original | Deduction | | |  | Addition | | Final |
| --- | --- | --- | --- | --- | --- | --- | --- | --- |
|  |  | Removed | Merged | Divided |  | Sub-divided | Added |  |
| Domain | 3 | 0 | 0 | 1 |  | 2 |  | 4 |
| Sub-domain | 15 | 1 | 2 | 1 |  | 2 | 2 | 15 |
| Indicators | 44 | 3 | 11 | 4 |  | 9 | 12 | 47 |
